# The Mediterranean Diet and Age-Related Eye Diseases: A Systematic Review

**DOI:** 10.3390/nu15092043

**Published:** 2023-04-23

**Authors:** Yi Wu, Ye Xie, Yixiong Yuan, Ruilin Xiong, Yuxin Hu, Kang Ning, Jason Ha, Wei Wang, Xiaotong Han, Mingguang He

**Affiliations:** 1 State Key Laboratory of Ophthalmology, Zhongshan Ophthalmic Center, Sun Yat-sen University, Guangdong Provincial Key Laboratory of Ophthalmology and Visual Science, Guangdong Provincial Clinical Research Center for Ocular Diseases, Guangzhou 510060, China; wuyi68@mail2.sysu.edu.cn (Y.W.); yuanyx5@mail2.sysu.edu.cn (Y.Y.); xiongrl0918@163.com (R.X.); mingguang_he@yahoo.com (M.H.); 2 Zhongshan Medical School, Sun Yat-sen University, Guangzhou 510080, China; xiey235@mail2.sysu.edu.cn (Y.X.); victoria.0124.hyx@gmail.com (Y.H.); 3 Department of Head and Neck Surgery, Sun Yat-sen University Cancer Center, Guangzhou 510060, China; ningkang@sysucc.org.cn; 4 Collaborative Innovation Center for Cancer Medicine, State Key Laboratory of Oncology in Southern China, Guangzhou 510060, China; 5 Centre for Eye Research Australia, Royal Victorian Eye and Ear Hospital, Melbourne, VIC 3002, Australia; jasha8@gmail.com; 6 Ophthalmology, Department of Surgery, University of Melbourne, Melbourne, VIC 3010, Australia

**Keywords:** Mediterranean diet, age-related eye diseases, age-related macular degeneration, dry eye syndrome, cataract, glaucoma, diabetic retinopathy

## Abstract

The Mediterranean diet (MD) is a healthy diet pattern that can prevent chronic age-related diseases, especially age-related eye diseases (AREDs) including cataract, glaucoma, age-related macular degeneration (AMD), diabetic retinopathy (DR) and dry eye syndrome (DES). In this study, we systematically reviewed studies in the literature that had reported associations between adherence to the MD and the five above-mentioned AREDs. Randomized controlled trials as well as prospective and retrospective observational studies were included; 1164 studies were identified, of which 1, 2, 9, 2 and 4 studies met our eligibility criteria for cataract, glaucoma, AMD, DR, and DES, respectively. According to these studies, higher MD adherence was associated with reduced risks of incident DR, incident AMD and progression to late AMD, but whether early and neovascular AMD could be alleviated remained to be debated. The results regarding the effects of the MD on DES were mixed, with three studies reporting an associations between MD and decreased severity or incidence of DES, whereas one study reported the opposite. No significant associations were observed between the MD and cataract or glaucoma. Generally, convincing evidence suggested a protective effect of the MD against AMD and DR. However, the evidence for cataract, glaucoma, and DES was less conclusive, and high-quality studies are needed for comprehensive evaluations of the potential benefits of MD on these eye diseases.

## 1. Introduction

Vision is a key part of overall health which affects people’s psychological, emotional and social well-being [1]. On the basis of estimates from the Global Burden of Disease Study (GBD), in 2020, around 295 million people had moderate to severe visual impairment (VI) and 43.3 million people were blind worldwide [2]. The leading causes of blindness and low vision are primarily age-related eye diseases (AREDs) including cataract, age-related macular degeneration (AMD), glaucoma and diabetic retinopathy (DR) [3]. With the rapid aging of the global population, a significant rise in the population with VI and blindness caused by AREDs or other eye conditions such as dry eye syndrome (DES) will be witnessed [3,4]. This escalating burden is expected to pose substantial healthcare and economic challenges for individuals as well as society at large. Therefore, it is important to identify risk factors for AREDs and to develop targeted interventions.

In addition to pharmacological and surgical approaches for clinical treatment of AREDs, dietary intervention is emerging as a more cost-effective and broadly applicable method for eye care [5]. The Mediterranean diet (MD) is potentially one of the best dietary patterns regarding health benefits and sustainability, and it has been shown to be strongly associated with increased longevity and decreased risk of chronic age-related pathologies including cancer [6], dementia [7] and cardiovascular diseases [8]. Existing definitions of the MD differed slightly, but all emphasized similar components: high consumption of plant-based food (vegetables, nuts, cereals, fruits and beans) and olive oil as the primary sources of dietary fat; low-to-moderate intakes of dairy products, poultry, red wine and fish; low intakes of red meat and sweets [9]. Considering the synergistic effects and interactions among different ingredients, the MD as a whole rather than single components is more likely to be responsible for the beneficial effects. 

In recent decades, there has been growing interest in investigating the effects of the MD on eye diseases. The protective effects of the MD against late AMD and DR have been suggested in several previous studies [10,11,12,13,14]. Omega-3 (n-3) PUFA, as one of the nutrients enriched in the MD, was reported to alleviate DES by decreasing proinflammatory prostaglandin in lacrimal gland [15] and rehabilitating meibomian gland function [16]. In addition, lutein, zeaxanthin, eicosapentaenoic acid and docosahexaenoic acid, which are also abundant in the MD, have been correlated with decreased serum concentrations of C-reactive protein, suggesting a potential role in lowering systemic inflammation in patients with AMD [17,18]. The MD could improve metabolic syndrome and obesity which are all closely associated with AREDs [19]. However, conflicting results have emerged regarding the relationship between the MD and the incidence of early-stage AMD [20,21]. Moreover, correlations between the MD and other AREDs, such as cataract and glaucoma, have received limited attention and have not been fully summarized and discussed. Hence, this study was conducted to provide a systematic literature review regarding the relationship between MD and five common AREDs.

## 2. Methods

### 2.1. Protocol Registration

The present systematic review adhered to the Preferred Reporting Items for Systematic Reviews and Meta-analyses (PRISMA) guidelines [22], ensuring a comprehensive and transparent assessment of the available studies. The protocol of this systematic review has been registered with the International Prospective Register of Systematic Reviews (http://www.crd.york.ac.uk/PROSPERO, accessed on 24 October 2022, registration no. CRD 42022367026), providing a detailed plan for the conduct and reporting of the review. 

### 2.2. Search Strategy

Electronic databases including PubMed and Embase were thoroughly searched, from the beginning of their inception until 20 February 2023. The keywords included ‘Mediterranean diet’, ‘cataract’, ‘glaucoma’, ‘retinal degeneration’, ‘diabetic retinopathy’, ‘dry eye syndrome’, ‘age-related eye diseases’. Further details of the searching strategy are shown in Supplementary Tables S1 and S2.

### 2.3. Eligible Criteria

Studies were included if they met the following criteria: (1) randomized controlled trials (RCTs), prospective and retrospective observational studies; (2) reported associations between MD adherence or MD intervention; (3) major AREDs (cataract, glaucoma, AMD, DR and DES) in human adults; (4) published in English. MD adherence in observational studies needed to be measured by standardized and validated tools, such as mediSCORE, the original Mediterranean Diet Score (MDS) developed by Trichopoulou et al. [23], and the alternative Mediterranean diet score (aMED) which is a variant of the MDS. The AREDs also needed to be identified based on the medical records or clinical ophthalmic examinations. Exclusion criteria were as follows: (1) non-original research such as case reports, reviews, letters, comments and meta-analyses; (2) studies that examined single nutrients or food components individually instead of taking the MD as a whole; (3) studies that only reported combined association of the MD and other lifestyles (e.g., physical exercise) with AREDs; (4) studies that lacked sufficient information to assess the impact of the MD on AREDs. Unpublished data and those studies were also excluded if they were not published in a peer-reviewed publication. 

### 2.4. Study Selection and Data Extraction

After removing duplicates, all titles and abstracts underwent a preliminary screening. Full-text articles of potentially relevant studies were retrieved for further assessment of eligibility by two independent reviewers (Y.W. and Y.X.). Any discrepancies were addressed through discussions with a senior reviewer (W.W.). Additionally, manual reference list searches of retrieved studies were conducted to identify additional eligible studies.

Data extraction was conducted according to a predefined data extraction form by two reviewers (Y.W. and X.H.) independently, and the results were further verified by a senior reviewer (W.W.). The following information was extracted from each study: first author and publication year, study design, study population, sample size, outcome of interest, exposure assessment, main findings and adjusted confounders. In cases where several risk estimates were reported with adjustments for different confounders, we prioritized the result based on the sample size. The reviewers came to an agreement on any discrepancies in data extraction.

### 2.5. Study Quality Assessment

The Newcastle-Ottawa Quality Assessment Scale (NOS) was employed to evaluate the quality of case-control and cohort studies based on three domains: selection of study groups (0–4 scores), comparability of groups (0–2 scores) and ascertainment of exposure or outcomes (0–3 scores) [24,25]. The total scores of 0–3, 4–6 and 7–9 were considered to represent low, moderate and high quality, respectively. The Agency for Healthcare Research and Quality (AHRQ) methodological checklist was used to assess the quality of cross-sectional studies [26]. The AHRQ methodological checklist contains 11 items, and each item received a score of 0 (answered “no” or “unclear”) or 1 (answered “yes”). The overall scores of 0–3, 4–7, and 8–11 were categorized as low, moderate, and high quality, respectively. To evaluate the quality of RCTs, the Cochrane risk of bias tool was employed, which assesses six domains, including sequence generation, allocation concealment, binding, incomplete outcome data, selective outcome reporting and other bias [27]. The studies were categorized as high, low or unclear risk of bias in each domain. Two investigators (Y.W. and Y.X.) independently evaluated the studies, and disagreements were resolved by consensus.

## 3. Results

### 3.1. Study Selection

**Figure 1:**
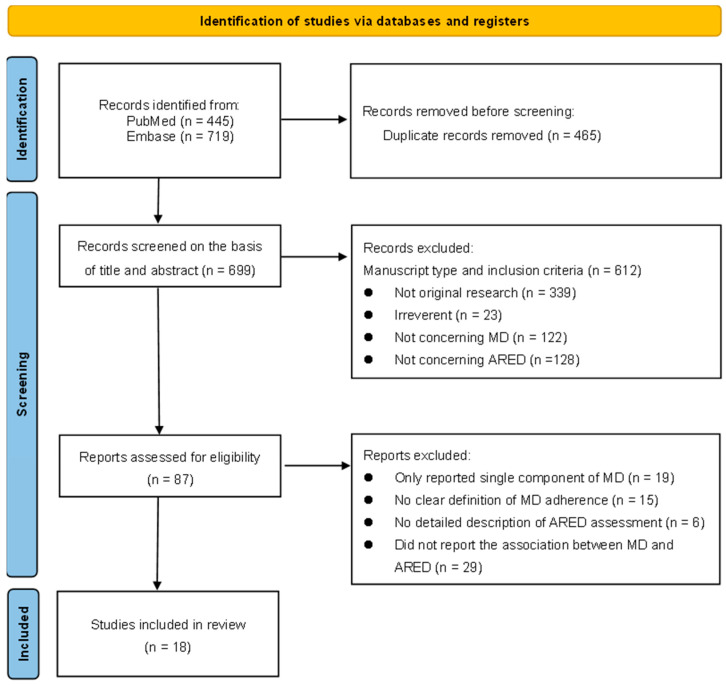
Flow diagram of screening studies. Abbreviations: MD, Mediterranean diet; AREDs, age-related eye diseases.

After initial searching, we identified 1164 possibly relevant studies from the electronic databases. After removal of 465 duplicates, 699 studies were screened based on the abstract and title. Six hundred and twelve studies were excluded for various reasons (not original research (*n* = 339); irreverent (*n* = 23); not concerning the MD (*n* = 122); not concerning AREDs (*n* = 128). Then, 87 studies were further excluded in full-text screening, of which 29 studies were without detailed information about the association between the MD and AREDs, 6 studies were without a detailed description of ARED assessment, 15 studies were without a clear definition of MD adherence, and 19 studies only reported on a single component of the MD. In total, 18 studies met our eligibility requirements and were eventually included in this systematic review [10,11,12,13,14,20,21,28,29,30,31,32,33,34,35,36,37,38] (Figure 1).

### 3.2. Baseline Characteristics

Among the 18 included studies, there were five prospective cohort studies [12,13,14,32,37], two retrospective cohort studies [10,11], four case-control studies [28,29,35,38], four cross-sectional studies [20,21,33,34] and three RCTs [30,31,36]. These studies were subdivided into cataract (*n* = 1) [30], glaucoma (*n* = 2) [37,38], AMD (*n* = 9) [10,11,12,13,14,20,21,28,29], DR (*n* = 2) [31,32] and DES (*n* = 4) [33,34,35,36]. The majority of the studies were performed in Europe (*n* = 11) [13,21,28,29,30,31,33,35,36,37,38], followed by USA (*n* = 6) [10,11,12,14,20,34] and Asia (*n* = 1) [32]. The median follow-up duration of cohort studies was 9.0 years (range 3.1–12 years) and the median follow-up duration of RCTs was 5.85 years (range 5.7–6 years). The median sample size was 2373 participants (range 34–22,187 participants). Two of these studies were limited to either females or males [20,34] (Table 1).

### 3.3. Assessment Method of MD Adherence

Amnog the 18 included studies, MD adherence was assessed using several scoring systems. The original alternate Mediterranean diet score (aMED score) and the Mediterranean score (mediSCORE) were applied in three [12,14,20] and four [13,28,29,34] studies, respectively. Two studies used a modified aMED score [10,11] or modified mediSCORE [35,37], respectively. There were specific modified MD scores (adopted by Martinez-Gonzalez et al. [39], Schroder et al. [41] and Ikram [40], respectively) adopted in three studies. The 14-item PREvencion con DIeta MEDiterranea (PREDIMED) tool and the 28-item Mediterranean Lifestyle (MEDLIFE) index were used in one study [33]. One of the studies used biological compliance markers (urine hydroxytyrosol levels and plasma alpha-linolenic acid proportions) to assess MD adherence [31]. The MD score was included as a continuous, binary, tripartite or quadripartite variable in non-RCT studies.

### 3.4. Diagnosis and Grading Method of ARED

The study outcome of ARED was measured according to several relevant criteria. One cataract study utilized both a medical diagnosis and surgical records [30]. One glaucoma study applied a self-reported diagnosis of glaucoma [37], and another study used eye examination [38]. For AMD, all nine studies [10,11,12,13,14,20,21,28,29] determined the occurrence of AMD based on eye examinations and color fundus photography. In addition, various grading systems were employed to evaluate the AMD stages, including the Wisconsin Age-Related Maculopathy Grading System (WARMGS) [11,20], a modified WARMGS [13], the International Classification System (ICS) [21,28,29], the Clinical Age-related Maculopathy Staging (CARMS) [14] and a distinct grading proposed by Merle et al. [12]. For DR, one study used the specific codes from the International Classification of Diseases, Tenth Revision (ICD-10) [32], and another study used medical diagnosis and clinical records of laser photocoagulation treatment [31]. All four studies of DES used eye examinations for its severity and incidence [33,34,35,36].

### 3.5. MD and AMD

Most studies that investigated the relationship between the MD and AREDs focused on AMD, including two cross-sectional studies [20,21], two case-control studies [28,29], three prospective cohort studies [12,13,14] and two retrospective studies [10,11]. In general, the protective effects of MD against AMD were confirmed. While most studies reported a decrease in AMD incidence and alleviation of AMD progression, there were discrepancies in the specific aspects of AMD (e.g., geographic atrophy, neovascular AMD and large drusen) that were improved by the MD.

Four studies discussed a correlation between the MD and the incidence or prevalence of AMD [20,21,28,29]. Two case-control studies (Nune et al. and Raimundo et al.) found that higher MD adherence (mediSCORE ≥ 6) was associated with a lower incidence of developing AMD, with a decreased incidence of 27% and 38%, respectively [28,29]. In two cross-sectional studies, Mares et al. reported a significant association between a higher aMED score and lower prevalence of early AMD [20], while a significant correlation was not found in the study carried out by Hogg et al. [21]. One study showed a significant association between higher MD adherence and a lower prevalence of neovascular AMD [21].

The other five studies all reported that higher MD adherence reduced the risk of AMD progression (defined as progression to advanced AMD, including geographic atrophy, neovascular AMD and large drusen, or biomarker progression such as drusen size and geographic atrophy enlargement) [10,11,12,13,14]. Advanced AMD risk was reduced by 22%, 26%, and 47% in studies by Keenan et al. (2020), Merle et al. (2015), and Merle et al. (2019), respectively [11,13,14]. Additionally, the risk of drusen size progression was reduced by 17% in the higher MD score group (aMED 4–9) compared to the lower MD score group (aMED 0–3) [12]. In a subgroup analysis of AMD progression, Agrón et al. found that there was a significant correlation between higher aMED and slower enlargement of geographic atrophy [10], and Keenan et al. also reported significant associations between mediSCORE and geographic atrophy, neovascular AMD as well as large drusen [11]. However, in another study, only atrophic AMD, but not neovascular AMD, was proven to be significantly associated with mediSCORE [13]. 

### 3.6. MD and DES

Three studies, including one RCT, showed that the MD could relieve DES symptoms [33,35,36], while the MD was identified as a risk factor for DES in another study [34]. Molina-Leyva et al. conducted an RCT on patients with metabolic syndrome in Spain and found that MD intervention relieved dry eye, as evidenced by improved DESS scores (−0.35 ± 0.15, *p* = 0.025) and OSDI scores (−1.75 ± 0.9, *p* = 0.039) [36]. In addition, a study of primary Sjögren’s syndrome patients found an inverse correlation between the PREDIMED score and disease activity as measured by ESAID (Spearman’s rho = −0.027, *p* = 0.009) and ClinESSAID (Spearman’s rho = −0.026, *p* = 0.01) [33]. In a study that compared 82 cases to 51 non cases, Machowicz et al. demonstrated that a higher MD score was linked to a lower risk of primary Sjögren’s syndrome [35]. Conversely, Galor et al. reported that the risk of developing or worsening dry eye symptoms was elevated by higher adherence to MD (*n* = 247; *p* = 0.03) [34]. However, this association disappeared after removing alcohol, vegetables, legumes and fish from the score, suggesting that certain dietary habits in the MD may be risk factors for dry eye syndrome.

### 3.7. MD and Other AREDs (Cataract, Glaucoma and DR)

To date, only one study has investigated the relationship between the MD and cataract, with negative results [30]. In an RCT conducted by García-Layana et al., 5802 elderly participants were assigned to one of the following three intervention groups: MD enriched with extra-virgin olive oil, MD enriched with nuts or a recommended low-fat diet. The incidence of cataract surgery among the three groups did not significantly differ after a 7 year follow-up period.

This review identified two studies that discussed the association between the MD and glaucoma, both of which found no significant association [37,38]. One perspective study conducted by Moreno-Montañés focused on the association between glaucoma and a Mediterranean lifestyle (including no smoking, Mediterranean diet and physical activity). A Mediterranean lifestyle score was used to divide the participants into four groups. After a median follow-up of 12 years, individuals in the highest Mediterranean lifestyle score group showed a lower incidence of glaucoma compared with the lowest score group, whereas, no significant link between the MD and glaucoma was found in a separate analysis of each Mediterranean lifestyle. Similarly, in a case-control study, Vergroesen et al. revealed no significant link between odds of the open-angle glaucoma and MD adherence [38]. The significant association was only observed between adherence to the Mediterranean-DASH Intervention for Neurodegenerative Delay (MIND) diet and glaucoma.

There were two studies that investigated the association between DR incidence and MD adherence, including one prospective study [32] and one RCT [31]. A post hoc analysis of the PREDIMED study demonstrated that participants with T2DM achieved a 40% reduction in DR incidence in the MD group (either added mixed nuts or EVOO) as compared with the control group [31], and the MD with added EVOO manifested greater protection on DR incidence than the MD with added nuts (−44% vs. −37%, *p* < 0.001)). In addition, Ghaemi et al. found that the MD diminished the risk of developing retinopathy in both T1DM and T2DM patients (−68% and −32%, respectively, *p* < 0.001) [32].

### 3.8. Quality Control Score

**Figure 2:**
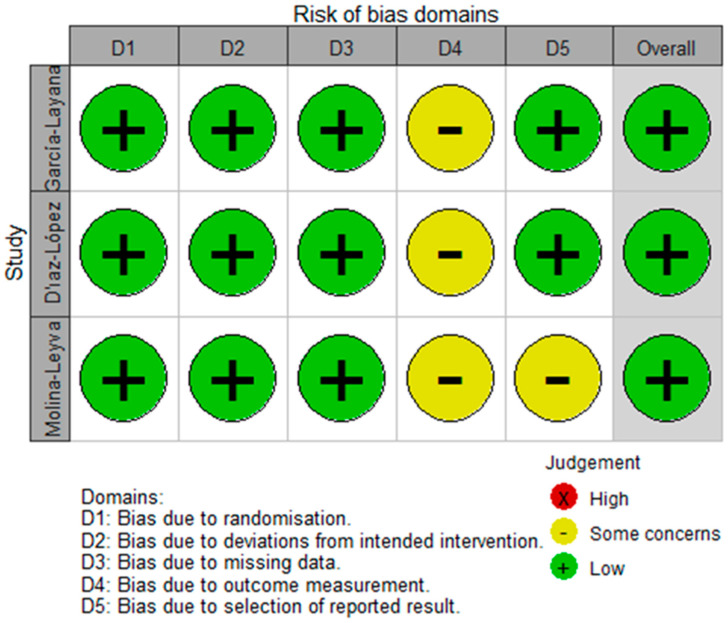
Risk of bias summary: Each risk of bias item for the included RCTs.

The methodological quality of the cohort and case-control studies [10,11,12,13,14,28,29,32,35,37,38] (*n* = 11) ranged from moderate to high with a mean NOS score of 7.3 (range 6–9) (Supplementary Tables S4 and S5). The quality of cross-sectional studies [20,21,33,34] (*n* = 4) was considered to be moderate with a mean AHRQ score of 6 (range 4–8) (Supplementary Table S6). All three RCT studies [30,31,36] were rated as having a low risk of bias for random sequence generation, allocation concealment and blinding, and were rated as having an unclear risk of bias for incomplete outcome data (Supplementary Table S3). Concerning selective outcome reporting, two RCTs [30,31] were rated as having a low risk of bias, while the remaining RCT [36] was judged as having an unclear risk of bias due to insufficient information. Additionally, no other potential bias sources were identified within all included studies. No studies were classified as having a high risk of overall bias, which is defined as having high or unclear risk of bias in at least three domains of the risk of bias tool (Figure 2).

## 4. Discussion

Given the ever-increasing population with AREDs and their significant impacts on public and individual health and economy [1], it is crucial to explore modifiable risk factors and interventions for these conditions. The MD, known for its health benefits and sustainability, has been extensively researched to reduce the risk of multiple chronic age-related pathologies. While the link between the MD and AMD has been well investigated, its correlation with other AREDs such as cataract, glaucoma and DR is less clear. In this systematic review, we summarized results from 18 studies, including nine studies on AMD, one study on cataract, two studies on DR, four studies on DES and two studies on glaucoma. Our findings indicate that most studies reported a significant association between the MD and reduced progression risk of AMD, but whether early or neovascular AMD could be alleviated by the MD is debateable. The included studies showed a protective role of the MD against incident DR, while a correlation between the MD and DES was controversial. No protective relationship between the MD and glaucoma or cataract was found, but the paucity of studies in these areas suggests caution when interpreting negative results.

The MD has been associated with numerous health benefits, including reducing the risk of several chronic diseases such as cardiovascular disease [8], type 2 diabetes [42], certain types of cancer [6] and neurodegenerative diseases [7]. There are several potential mechanisms by which the Mediterranean diet can improve various diseases including AREDs. The MD has been shown to increase the intake of protective nutrients, such as vitamins, unsaturated fatty acids, dietary fiber and minerals, while decreasing the intake of proinflammatory foods such as trans fatty acids and refined sugar [43]. The MD also could promote the development of favorable gut bacteria and could help to regulate blood sugar levels and improve insulin sensitivity [43]. 

The protective role of the MD in delaying the onset and progression of AMD has been reported in a previous review [44], but the underlying mechanism remains to be controversial. Antioxidants enriched in the MD have been considered to be protective components against the onset of AMD [45]. Additionally, the MD has reportedly been shown to have a preventive impact on obesity-related pathologies, which may contribute to AMD through adipocytokines affecting the function of pigment epithelial cells [46]. Although an association between the MD and AMD progression has been clearly demonstrated, there is controversy regarding which aspect of AMD progression is reduced by the MD (geographic atrophy or neovascular AMD). While it has been reported that the MD reduced the odds of neovascular AMD [11,21], Merle et al. reported negative results [13]. The hereditary susceptibility among people with different ethnical backgrounds may explain this controversy. For instance, people with rs10922109 alleles showed a lower risk of geographic atrophy than neovascular AMD when following the MD; however, the frequency of the rs10922109 alleles in the included population may have affected the results of the study [11]. Since most existing studies have focused on Caucasian populations, the generalizability of the findings to all populations is limited, and more studies are needed for Asian and African populations.

The MD was reported to reduce DR incidence due to its dietary relevance. Many vegetables, seeds and fruits in the MD could combat inflammation, oxidative stress and insulin resistance, which are pathogenic factors in diabetes and its complications [47]. Omega-3 fatty acids, which are abundant in the MD, contribute significantly to the neuroprotective and anti-inflammatory effects, which could protect diabetic patients from nerve and microvascular lesions [48]. PREDIMED studies reported that with a protective intake of omega-3 fatty acids, the risks of DR and metabolic syndrome would be decreased in diabetic patients [49]. In addition, omega-3 fatty acids in proliferative diabetic retinopathy were conducive to improving diabetic macular edema and avoiding neovascularization [50]. However, there are also some defects in the PREDIMED studies of DR. For example, there were missing data on glycated hemoglobin, and the RCTs only recruited elderly people who are vulnerable to cardiovascular disease [31]. Thus, studies with more comprehensive data on DR are needed in the future. 

The impact of the MD on DES was inconclusive. Three studies [33,35,36] showed a beneficial effect of the MD on dry eye, possibly due to n-3 PUFAs that are enriched in fish and olive oil, which are both recommended in the MD. However, Galor et al. [34] demonstrated that the MD showed a harmful impact on either dry eye incidence or disease severity, while this adverse correlation disappeared after the removal of alcohol from analysis. In this study, moderate alcohol recommended by the MD resulted in a two-fold increase in the odds of developing dry eye disease and worse meibomian gland function. The controversy mainly focuses on whether alcohol is completely harmful to patients with dry eye even with a moderate intake (refers to >0 drinks per week but ≤2 drinks per day). For example, a meta-analysis demonstrated that any level of alcohol consumption increased the odds (OR = 1.33, 95% CI, 1.31–1.34) of suffering from dry eye syndrome compared to non-drinkers [51]. Therefore, when recommending the MD as a healthy lifestyle, consideration should be given to reduce alcohol intake to nil. It has also been suggested that the MD and its scoring rule could be modified for different patient populations if necessary.

The available data do not reveal an association between the MD and cataract development [30], which may be due to several limitations. The studies were only conducted in patients with type 2 diabetes or cardiovascular risk factors, which accelerated the progression of cataract by the production of proinflammatory cytokines, potentially counteracting the protective effect of MD. Additionally, earlier cataract extraction in patients with diabetes or the low-fat diet in the control group could mask the benefits of the MD. Furthermore, using cataract surgery as the endpoint may limit the sensitivity of detecting the development of cataract. Given the scarcity of existing studies, further research in healthy populations with more sensitive outcome measures is an unmet need to investigate the association.

The impact regarding the MD on glaucoma remains inconclusive based on the existing studies. The prospective study included in the analysis relied on self-reported diagnosis and dietary patterns, which may have led to an overestimation of MD adherence, as well as missing undiagnosed cases of glaucoma [37]. Additionally, in their model, they adjusted calorie intake, caffeine intake, alcohol intake and the omega-3/omega-6 ratio for the result, while these may mediate the effect of MD on incident glaucoma. Similarly, the case-control study had limitations due to under- or overestimation [38]. For these reasons, we should be more prudent when looking at this negative result. Given studies indicating that oxidative stress and some oxygen free radicals are involved in the formation of glaucoma [52], we remain confident that possible associations in the future may be found. Some studies have showen that chronic and low-grade inflammation also had an effect on the pathogenesis of glaucoma [53]. Therefore, given the anti-inflammatory and antioxidant effects of the MD, we believe that the MD would have some influence on the pathology pathway of glaucoma, which needs further investigation. The MIND diet, which separates green leafy vegetables from other vegetables and assesses berries individually instead of fruits as a whole, could serve as a potential modification of the MD in glaucoma [38].

Several limitations should also be acknowledged in this review. Firstly, the included criteria were limited to English language studies, potentially leading to exclusion of non-English relevant data and publication bias. Secondly, the heterogeneity of the included studies was high, mainly manifested by different study designs, MD scoring criteria and ARED diagnosis criteria. Therefore, a meta-analysis could not be conducted in those studies, and secondary studies are expected in the future. However, compared to previous studies discussing single nutrients, the advantages of these studies lie in their analysis of the MD as a whole diet pattern, which can better reflect the comprehensive role of various nutrients and their cumulative or synergistic impact on ocular health.

## 5. Conclusions

In summary, numerous studies have highlighted the potential protective effects of the MD on the incidence and progression of AMD and the incidence of DR. Nonetheless, it remains unclear whether early and neovascular AMD could be alleviated by the MD, and the relationship between the MD and dry eye disease remains a topic of debate. Furthermore, there is a paucity of research exploring the role of the MD in the development of cataracts and glaucoma, which poses challenges in establishing a definitive association. Nevertheless, the available evidence strongly suggests that the MD may hold significant promise in the management and prevention of various AREDs. Thus, additional high-quality studies are warranted to confirm and explicitly elucidate the potential mechanisms involved in the potential benefits of the MD for AREDs. In conclusion, the current body of evidence supports the potential role of the MD as a dietary intervention for ocular health, but further investigation is needed to establish its full therapeutic potential.

## Figures and Tables

**Table 1:** Characteristics of included studies.

Disease	Author (Year)	Country	Size (Male)	Age (Years)	Study Design	Follow-Up (Years)	Outcome of Interest	Case Definition	Measurement Indexes	Risk Factor	Summary of Main Findings (Including Secondary Outcomes)	Quality
AMD	Mares (2011) [20]	USA	1313 (0)	55–74	Cross- sectional	-	Presence	stereoscopic fundus photographs	Wisconsin AMD Grading System	aMED (quartered)	Early AMD (OR = 0.34 [0.08–0.89], *p* = 0.046)	8
AMD	Merle (2015) [14]	USA	2525 (1124)	55–80	Prospective	8.7	Progression	retinal stereoscopic images	CARMS	aMED (trichotomous)	Advanced AMD (HR = 0.74 [0.61–0.91], *p* = 0.007)	9
AMD	Nunes (2018) [28]	Portugal	1992 (879)	>55	Case-control	-	Presence	digital mydriatic color fundus photograph	International Classification and Grading System	mediSCORE (dichotomous)	AMD (OR = 0.73 [0.58–0.93], *p* = 0.009)	6
AMD	Raimundo (2018) [29]	Portugal	883 (385)	>55	Case-control	-	Presence	digital mydriatic color fundus photograph	International Classification and Grading System	mediSCORE (dichotomous)	AMD (OR = 0.62 [0.38–0.97], *p* = 0.04)	7
AMD	Hogg (2017) [21]	Several European countries	2262 (1028)	>65	Cross- sectional	-	Presence	stereoscopic digitized color fundus images	International classification and grading system	MDS from Martinez-Gonzalez et al. (2004) [39] (quartered)	Neovascular AMD (OR = 0.53 [0.27–1.04], *p* = 0.01); large drusen (OR = 0.80 [0.65–0.68], *p* = 0.01); early AMD and atrophic AMD (negative)	7
AMD	Merle (2019) [13]	France & Neverland	4996 (2022)	RS-Ⅰ > 55 Alienor > 73	Prospective	RS-Ⅰ (9.9) Alienor (4.1)	Progression	fundus photographs	Modification of the Wisconsin Age-Related System and International Classification	mediSCORE (trichotomous)	Advanced AMD (HR = 0.53 [0.33–0.84], *p* = 0.009); atrophic AMD (HR = 0.42 [0.20–0.90], *p* = 0.04); neovascular AMD (negative)	6
AMD	Merle (2020) [12]	USA	1838 (1328)	55–80	Prospective	10.2	Progression	Retinal stereoscopic color photographs	Drusen size grade	aMED (dichotomous)	Drusen progression (HR = 0.83 [0.68–0.99], *p* = 0.049);	9
AMD	Keenan (2020) [11]	USA	7756 (4385)	55–80	Retrospective	10.2	Progression	Color fundus photographs	Wisconsin AMD Grading System	Modified aMED (trichotomous)	Late AMD (HR = 0.78 [0.71–0.85], *p* < 0.0001); geographic atrophy (HR = 0.71 [0.63–0.80], *p* < 0.0001); neovascular AMD (HR = 0.84 [0.75–0.95], *p* = 0.005); large drusen (HR = 0.79 [0.68–0.93], *p* = 0.04)	8
AMD	Agrón (2022) [10]	USA	4203	50–85	Retrospective	3.1	Progression	Digital stereoscopic color fundus photographs	Square root of geographic atrophy area	Modified aMED (trichotomous)	Geographic atrophy enlargement (*p* = 0.008, T3; 0.256 mm/year [0.236–0.276], T2: 0.290 mm/year [0.268–0.311], T1: 0.298 mm/year [0.280–0.317])	7
DES	Galor (2014) [34]	USA	258 (258)	>50	Cross-sectional	-	Presence	-	DES severity score	mediSCORE (continuous)	DES (OR, 1.25 [1.06–1.47], *p* = 0.007) and increased disease severity of DES (*p* = 0.03)	5
DES	Leyva (2020) [36]	Spain	34 (13)	M (55–75) F (60–75)	RCT	-	Dry eye parameters and symptoms	External eye examination	Dry Eye Scoring System; Ocular Surface Disease Index	MD intervention	Relieved dry eye symptoms in DESS test score, −0.35 ± 0.15 (*p* = 0.025) and OSDI, −1.75 ± 0.9 (*p* = 0.039)	*
DES	Carubbi (2021) [33]	Italia	93 (5)	61.8 (mean)	Cross-sectional	-	-	-	ESSDAI and ESSPRI	PREDIMED, MEDLIFE (continuous)	Decreased disease activity measured by ESSDAI (Spearman’s rho = −0.27, *p* = 0.009) and ClinESSDAI (Spearman’s rho = −0.26, *p* = 0.01)	4
DES	Machowicz (2020) [35]	UK	133 (9)	>18	Case-control	-	Presence	-	EULAR and ESSDAI	Modified mediSCORE (continuous)	pSS (OR = 0.81 [0.66–0.99], *p* = 0.038)	6
Cataract	Layana (2017) [30]	Spain	5802 (2598)	M (55–80); F (60–80)	RCT	5.7	Incidence	Surgery medical records	-	MD intervention (vs. low-fat diet)	Cataract surgery (negative)	*
Glaucoma	Montañés (2022) [37]	Spain	18,420 (7332)	37.7 (mean)	Prospective	12	Incidence	Self-reported diagnosis by ophthalmologist	-	Modified mediSCORE excluding alcohol; SHLS (quartered)	Glaucoma of MD only (negative) and glaucoma of SHLS (HR = 0.51 [0.28–0.93])	8
Glaucoma	Vergroesen (2023) [38]	Neverland	1020 (468)	>45	Case-control	5	Presence	Eye examination	-	MDS from Ikram et al. 2020 (continuous) [40]; MIND diet	OAG of MD (nagative) and OAG of MIND (OR = 0.80 [0.66–0.96], *p* = 0.02)	6
DR	D’ıaz-López (2015) [31]	Spain	3614 (1707)	M(55–80); F(60–80)	RCT	6.0	Incidence	Nonmydriatic fundus camera	-	MD intervention (vs. low-fat diet), Biomarkers (urine hydroxytyrosol, a-linolenic acid) (quintiled)	DR for the MedDiet + EVOO (HR = 0.56 [0.32–0.97]) and the MedDiet + nuts (negative); DR for adherence to MD measured by biomarkers (HR, 0.34 [0.13–0.89]; *p* = 0.001)	*
DR	Ghaemi (2021) [32]	Iran	22,187 (6705)	T1D (50.7) T2D (59.9) (mean)	Prospective	2–11	Incidence	-	International Classification of Diseases	MDS from Schroder et al. 2011 [41] (dichotomous)	DR in T1DM (OR = 0.32 [0.24–0.44], *p* < 0.001) and DR in T2DM (OR = 0.68 [0.61–0.71], *p* < 0.001)	8

Abbreviations: AMD, age-related macular degeneration; Alienor, Antioxydants, Lipides Essentiels, Nutrition et Maladies Oculaires study; aMED, alternative or alternate Mediterranean diet score; AREDS/AREDS2, Age-Related Eye Disease Study; BMI, body mass index; CAREDS, Carotenoids in Age-Related Eye Disease Study; CARMS, clinical age-related maculopathy staging; DES, dry eye syndrome; DESS, dry eye scoring system; DR, diabetic retinopathy; EUREYE, European Eye (study); ESSDAI, EULAR Sjögren’s syndrome disease activity index; ESSPRI, EULAR Sjögren’s syndrome patient reported index; EULAR, European League Against Rheumatism; FFQ, Food Frequency Questionnaire; HR, hazard ratio; ICS, International Classification System; MDS, Mediterranean diet score; mediSCORE, Mediterranean score; MEDLIFE, Mediterranean Lifestyle index; MIND, Mediterranean-DASH Intervention for Neurodegenerative Delay; OAG, open-angle glaucoma; OR, odds ratio; OASIS, Optimising Assessment in Sjögren’s Syndrome; OSDI, ocular surface disease index; PREDIMED, PREvención con DIeta MEDiterránea; Pss, primary Sjögren’s syndrome; RCT, randomized controlled trial; RS-I, Rotterdam Study I; SHLS, SUN Healthy Lifestyle Score; WARMGS, Wisconsin Age-Related Maculopathy Grading System; WHIOS, Women’s Health Initiative Observational Study. * RCT quality (Supplementary Table S3).

## Data Availability

Not applicable.

## Supplementary Materials

The following supporting information can be downloaded at: https://www.mdpi.com/article/10.3390/nu15092043/s1, Table S1: The search strategy of PubMed used in this systematic review (From inception to 20 February 2023); Table S2: The search strategy of Embase used in this systematic review (From inception to 20 February 2023); Table S3: The quality of included randomized control trial studies assessed by the Cochrane Risk of Bias Tool; Table S4: The quality of included cohort studies assessed by the Newcastle-Ottawa Quality Assessment Scale; Table S5: The quality of included case-control studies assessed by the Newcastle-Ottawa Quality Assessment Scale; Table S6: The quality of included cross-sectional studies assessed by the Agency for Healthcare Research and Quality methodology checklist *.
