# Supplementary material for: The Mediterranean Diet and Age-Related Eye Diseases: A Systematic Review
Source: Nutrients. 2023 Apr 23;15(9):2043. doi: 10.3390/nu15092043 (PMC10181476; doi:10.3390/nu15092043)
Supplement: Supplementary file 1 [file nutrients-15-02043-s001.zip › nutrients-2321431-supplementary.pdf]

Table S1. **The search strategy of PubMed used in this systematic review (From inception to Feb 20, 2023)**

PubMed		Number
#1	Diet, Mediterranean[MeSH Terms]	5071
#2	"Mediterranean diet*" OR "Mediterranean dietary pattern" OR "Mediterranean lifestyle" OR MedDiet	8412
#3	#1 OR #2	8412
#4	Cataract[Mesh Terms]	32293
#5	cataract OR lens opaci* OR capsule opaci*	86856
#6	(Glaucoma[Mesh Terms]) OR (Ocular Hypertension[Mesh Terms])	61701
#7	glaucoma OR "ocular hypertension" OR poag OR oag OR oht	87227
#8	(Retinal Degeneration[Mesh Terms]) OR (Retinal Neovascularization[Mesh Terms]) OR (Choroidal Neovascularization[Mesh Terms])	55884
#9	maculopath* OR macula* degener* OR macula* neovasc* OR macula* dystroph* OR macula* atroph* OR macula* edema* OR "geographic atroph*" OR drusen* OR amd OR armd	61514
#10	retina* degener* OR retina* neovasc* OR choroid* neovasc* OR cnv	64133
#11	Diabetic Retinopathy[Mesh Terms]	28952
#12	diabet* retinopath* OR proliferat* retinopath* OR diabet* macula* edema* OR diabet* complication* OR dr OR dme	679748
#13	(Dry Eye Syndromes[Mesh Terms]) OR (Meibomian Gland Dysfunction[Mesh Terms])	22577
#14	"dry eye" OR "ocular surface disease" OR "meibomian gland dysfunction" OR "Sjogren's syndrome" OR xerophthalmia OR "keratoconjunctivitis sicca"	32547
#15	OR #4-#14	940699
#16	#3 AND #15	523
#17	#16 filters: humans, English	445

Table S2. **The search strategy of Embase used in this systematic review (From inception to Feb 20, 2023)**

Embase		Number
#1	'Mediterranean diet'/exp	11589
#2	'Mediterranean diet*' OR 'Mediterranean dietary pattern' OR 'Mediterranean lifestyle' OR MedDiet	13809
#3	#1 OR #2	13859
#4	'Cataract'/exp	71254
#5	cataract OR 'lens opaci*' OR 'capsule opaci*'	120136
#6	'Glaucoma'/exp OR 'Ocular Hypertension'/exp	107565
#7	glaucoma OR 'ocular hypertension' OR poag OR oag OR oht	122412
#8	'Retinal Degeneration'/exp OR 'Retinal Neovascularization'/exp OR 'Choroidal Neovascularization'/exp	84593
#9	maculopath* OR macula* degener* OR macula* neovasc* OR macula* dystroph* OR macula* atroph* OR 'geographic atroph*' OR drusen* OR amd OR armd	37287
#10	retina* degener* OR retina* neovasc* OR choroid* neovasc* OR cnv	44774
#11	'Diabetic Retinopathy'/exp	54070
#12	diabet* retinopath* OR proliferat* retinopath* OR diabet* macula* edema* OR diabet* complication* OR dr OR dme	1022244
#13	'Dry Eye Syndromes'/exp OR ' Meibomian Gland Dysfunction'/exp	34406
#14	'dry eye' OR 'ocular surface disease' OR 'meibomian gland dysfunction' OR 'sjogren syndrome' OR 'xerophthalmia' OR 'keratoconjunctivitis sicca'	34986
#15	OR #4-#14	1398923
#16	#3 AND #15	786
#17	#16 AND [english]/lim	754
#18	#17 AND 'human'/de	719

Table S3. The quality of included randomized control trial studies assessed by the Cochrane Risk of Bias Tool

Reference	Sequence Generation	Allocation concealment	Blinding	Incomplete Outcome data	Selective Outcome Reporting	Other bias
García-Layana, 2017	low	low	low	unclear	low	low
D´ıaz-López, 2015	low	low	low	unclear	low	low
Molina-Leyva, 2020	low	low	low	unclear	unclear	low

Table S4. The quality of included cohort studies assessed by the Newcastle-Ottawa Quality Assessment Scale.

Category	Item	Option	Marle, 2015	Marle, 2019	Keenan, 2020	Marle, 2020	Ghaemi 2021	Agrón, 2022	Montañés, 2022
Selection	Representativeness of the exposed cohort	truly representative of the citizen that have Mediterranean diet the community *	N	N	N	N	N	N	N
		somewhat representative of the citizen that have Mediterranean diet in the community*	Y*	Y*	Y*	Y*	Y*	Y*	Y*
		selected group of users eg nurses, volunteers	N	N	N	N	N	N	N
		no description of the derivation of the cohort	N	N	N	N	N	N	N
	Selection of the non exposed cohort	drawn from the same community as the exposed cohort*	Y*	Y*	Y*	Y*	Y*	Y*	Y*
		drawn from a different source	N	N	N	N	N	N	N
		no description of the derivation of the non exposed cohort	N	N	N	N	N	N	N
	Ascertainment of exposure	secure record (e.g., surgical records)*	N	N	N	N	Y*	N	N
		structured interview*	Y*	Y*	Y*	Y*	N	Y*	N
		written self report	N	N	N	N	N	N	Y
		no description	N	N	N	N	N	N	N
	Demonstration that outcome of interest was not present at start of study	yes*	Y*	Y*	Y*	Y*	Y*	N	Y*
		no	N	N	N	N	N	Y	N
Comparability	Comparability of cohorts on the basis of the design or analysis	study controls for the most important factor*	Y*	N	Y*	Y*	Y*	Y*	Y*
		study controls for any additional factor (This criteria could be modified to indicate specific control for a second important factor.) *	Y*	Y*	Y*	Y*	Y*	Y*	Y*
Outcome	Assessment of outcome	independent blind assessment *	Y*	N	Y*	Y*	N	N	Y*
		record linkage *	N	N	N	N	Y*	Y*	N
		self report	N	N	N	N	N	N	N
		no description	N	Y	N	N	N	N	N
	Was follow-up long enough for outcomes to occur (NOT IN CROSS SECTIONAL)	yes, it is an adequate follow up period for outcome of interest*	Y*	Y*	Y*	Y*	Y*	Y*	Y*
		no	N	N	N	N	N	N	N
	Adequacy of follow up of cohorts (NOT IN CROSS SECTIONAL)	complete follow up - all subjects accounted for *	N	N	N	N	N	N	N
		subjects lost to follow up unlikely to introduce bias - small number lost - >80 % (select an adequate %) follow up, or description provided of those lost) *	Y*	N	N	Y*	N	N	Y*
		follow up rate < 80% (select an adequate %) and no description of those lost	N	Y	N	N	N	N	N
		no statement	N	N	Y	N	Y	Y	N
Quality Score			9	6	8	9	8	7	8

Table S5. The quality of included case-control studies assessed by the Newcastle-Ottawa Quality Assessment Scale.

Category	Item	Option	Nunes, 2018	Raimundo, 2018	Machowicz, 2020	Vergroesen, 2023
Selection	Is the case definition adequate?	Yes, with independent validation*	Y*	Y*	Y*	Y*
		Yes, for example, record linkage or based on self-reports	N	N	N	N
		No description	N	N	N	N
	Repetitiveness of the cases	Consecutive or obviously representative series of cases*	N	N	N	Y*
		Potential for selection biases or not stated	Y	Y	Y	N
	Selection of controls	Community controls*	Y*	Y*	N	Y*
		Hospital controls	N	N	Y	N
		No description	N	N	N	N
	Definition of controls	No history of disease*	Y*	Y*	Y*	N
		No description of source	N	N	N	Y
Comparability	Comparability of cases and controls on the basis of the design or analysis	Study controls for age*	N	Y*	Y*	N
		Study controls for any additional factor*	Y*	Y*	Y*	Y*
Exposure	Ascertainment of exposure	Secure record*	N	N	N	Y*
		Structured interview where blind to case/control status*	Y*	Y*	Y*	N
		Interview not blinded to case/control status	N	N	N	N
		Written self-report or medical record only	N	N	N	N
		No description	N	N	N	N
	Same method of ascertainment for cases and controls	Yes*	Y*	Y*	Y*	Y*
		No	N	N	N	N
	Non-Response rate	Same rate for both group*	N	N	N	N
		Non respondents described*	N	N	N	N
		Rate different and no designation	Y	Y	Y	Y
	Quality Score		6	7	6	6

Table S6. The quality of included cross-sectional studies assessed by the Agency for Healthcare Research and Quality methodology checklist*.

	Mares, 2011	Hogg, 2017	Galor, 2014	Carubbi, 2021
1) Define the source of information (survey, record review)	1	1	1	1
2) List inclusion and exclusion criteria for exposed and unexposed subjects (cases and controls) or refer to previous publications	1	1	1	1
3) Indicate time period used for identifying patients	1	1	0	0
4) Indicate whether or not subjects were consecutive if not population-based	1	1	0	1
5) Indicate if evaluators of subjective components of study were masked to other aspects of the status of the participants	0	1	0	0
6) Describe any assessments undertaken for quality assurance purposes (eg, test/retest of primary outcome measurements)	1	0	1	1
7) Explain any patient exclusions from analysis	1	0	0	0
8) Describe how confounding was assessed and/ or controlled	1	1	1	0
9) If applicable, explain how missing data were handled in the analysis	0	0	0	0
10) Summarize patient response rates and completeness of data collection	1	1	1	0
11) Clarify what follow-up, if any, was expected and the percentage of patients for which incomplete data or follow-up was obtained	0	0	0	0
Total scale	8	7	5	4

* Article quality was assessed as follows: low quality = 0-3; moderate quality = 4-7; high quality= 8 -11.
